# Leaky Partial Update LMS Algorithms in Application to Structural Active Noise Control

**DOI:** 10.3390/s23031169

**Published:** 2023-01-19

**Authors:** Dariusz Bismor

**Affiliations:** Department of Measurements and Control Systems, Silesian University of Technology, ul. Akademicka 16, 44-100 Gliwice, Poland; dariusz.bismor@polsl.pl

**Keywords:** partial updates, least mean squares, Leaky LMS, structural active noise control

## Abstract

Adaptive signal processing algorithms play an important role in many practical applications in diverse fields, such as telecommunication, radar, sonar, multimedia, biomedical engineering and noise control. Recently, a group of adaptive filtering algorithms called partial update adaptive algorithms (partial updates) has gathered considerable attention in both research and practical applications. This paper is a study of the application of PUs to very demanding, structural active noise control (ANC) systems, which are of particular interest due to their ability to provide for a global noise reduction. However, such systems are multichannel, with very high computational power requirements, which may be reduced by the application of partial updates. The paper discusses the modifications necessary to apply PUs in structural ANC systems and the potential computational power savings offered by this application. As a result, leaky versions of the PU LMS algorithms are introduced to the general public. The paper also presents two simulation examples, based on real laboratory setups, confirming high performance of the proposed algorithms.

## 1. Introduction

Many applications of adaptive filtering are computationally too expensive to be implemented in real time, even using modern hardware. Examples of such applications include wireless communication systems [1], radar systems [2], adaptive beamforming of radio signals [3], and active noise control [4,5]. Partial updates are an effective and modern way of reducing this computational effort [6,7]. Partial updates are particularly well-suited for those real-time applications that require a huge number of operations to be performed in a single sampling interval. For example, the Least Mean Square (LMS) algorithm applied to an acoustic echo cancellation and updating a finite impulse response (FIR) filter with *L* coefficients requires at least 2L multiply-and-accumulate (MAC) operations, *L* signal read, *L* coefficient read and *L* coefficient store operations [8]. Other applications, e.g., active noise control, can be even more expensive, computation-wise. For long adaptive FIR filters, these numbers may be too high to fit into one sampling period. For this and other reasons, there is a desire to develop and apply effective algorithms with a smaller number of operations.

Generally, the idea of partial updates can be applied to any iterative descent algorithm, e.g., Recursive Least Squares (RLS). However, in this publication, we will concentrate on the application of partial updates to the family of LMS algorithms. The purpose of this paper is to extend the idea of PU LMS algorithms over the group of LMS algorithms with leakage. To the best of our knowledge, a general analysis of such a combination has not been presented to the general public yet (except for the author’s local conference communications [5,9]). In the following chapter, the basic facts about the PU LMS algorithms are presented for the convenience of the reader (for an extended introduction and analysis, we recommend [6]). Then, the PU LMS algorithms with leakage are introduced in Section 3. Section 4 introduces one of the possible applications of such algorithms in the form of a multichannel active noise control system, and presents the results of simulations. Finally, conclusions are presented in Section 5.

## 2. Partial Update LMS Algorithms

The idea of partial updates is not new, as the first publications within this category are dated back to 1994 [10]. A good summary of scientific achievements concerned with PUs was presented in 2008 [6]. The purpose of this section is to present a short summary of PU LMS algorithms for the convenience of the reader.

There are two main groups of PU LMS algorithms: time-domain algorithms and transform-domain algorithms. This publication is concerned only with the first group. Time-domain PU LMS algorithms can be further divided into data-independent and data-dependent algorithms. An interesting observation is that while application of a data-independent algorithm always results in performance degradation (here we consider the performance to be a combination of the convergence speed and the final error level after a predefined period of time), the application of a data-dependent algorithm does not necessarily result in such degradation. Moreover, under some circumstances, it can even result in performance improvement [8]. On the other hand, data-independent algorithms offer the best computational power savings, requiring no additional operations to analyze the data.

Data-dependent PU algorithms analyze the input data to select those filter coefficients, which will result in the best filter improvement after the update. This operation usually involves sorting of the input vector u(n) (see below). Fortunately, computationally efficient sorting algorithms are now available, especially in cases when the input vector is constructed from a tapped-delay line, where only one vector element is exchanged in each sampling period [6,11].

The only disadvantage of data-independent PU LMS algorithms is slower convergence rate; therefore, those algorithms cannot be used in applications where fast convergence is critical. The level of convergence rate decrease depends on the number of an adaptive filter weights excluded from update. The disadvantage of data-dependent algorithms, on the other hand, is the necessity of implementation of the sorting (or finding the maximum). In this case, the number of parameters excluded from the update must be high enough to overcompensate the computational power of the sorting. This will probably be hard to achieve in case of short adaptive filters.

In this publication, we will focus our attention on the following, popular PU algorithms:

- Data-independent algorithms:
  − Periodic partial update LMS algorithm (periodic LMS),
  − Sequential partial update LMS algorithm (sequential LMS),
  − Sequential partial update NLMS algorithm (sequential NLMS),
  − Stochastic partial update LMS algorithm (stochastic LMS),
- Data-dependent algorithms:
  − M-max partial update LMS algorithm (M-max LMS),
  − M-max partial update normalized LMS algorithm (M-max NLMS),
  − Selective partial update (normalized) LMS algorithm (selective NLMS),
  One tap update LMS algorithm (OTU LMS).

The periodic LMS algorithm is probably the most intuitive of all: it updates all the filter coefficients once per *several* sampling periods. The properties and behavior of this algorithm are very similar to the full update LMS algorithm (except for the convergence rate); therefore, it will not be considered below.

The one tap update LMS algorithm is in fact the selective NLMS with the extreme selection of an update of a single filter tap only in every sampling period. It was enumerated separately due to a very important property: instead of sorting the whole input vector, this algorithm is based on finding the maximum absolute value of this vector. As searching for a maximum value is computationally more efficient than sorting [11], this algorithm offers the best computational power savings among all the data-dependent algorithms.

Before describing each of the above PU LMS algorithms, we will recall the (full update) Leaky LMS (LLMS) algorithm, which is given by [12]:

(1) w(n+1)=γw(n)+μ(n)u(n)e(n),

where γ is called the leakage factor and μ(n) is called the step size. The latter can be either constant ($\mu \left(n\right)=\tilde{\mu }$, historically the original LMS algorithm [13]) or variable. The filter tap vector and the input vector u(n) are given by:

(2) $$\begin{array}{cc} w(n) & ={\left[w_{0}\left(n\right)\;w_{1}\left(n\right)\;\ldots w_{L-1}\left(n\right)\right]}^{T}, \end{array}$$

(3) $$\begin{array}{cc} u(n) & ={\left[u(n)\;u(n-1)\;\ldots u(n-L)\right]}^{T}. \end{array}$$

Thus, the input vector contains samples from a single source of a signal, delayed in time—such a vector is usually referred to as the *tap-delayed vector*. As mentioned above, this specific structure can be utilized to sort the input vector more efficiently. For details, refer, e.g., to [11].

The error signal e(n) is usually calculated as:

(4) $$e\left(n\right)=d\left(n\right)-y\left(n\right)=d\left(n\right)-\sum_{l=0}^{L-1}w_{l}\left(n\right)u\left(n-l\right),$$

where d(n) is the desired signal, the values of which should be approximated by the filter output y(n) in a way that minimizes the expected value of the squared error.

## 3. PU LMS Algorithms with Leakage

The general equation describing PU LMS algorithms with leakage discussed in this paper, which applies to all but the periodic LMS, can be given as:

(5) $$w\left(n+1\right)=G_{M}\left(n\right)w\left(n\right)-\mu \left(n\right)I_{M}\left(n\right)u\left(n\right)e\left(n\right),$$

where $G_{M}\left(n\right)$ is the leakage matrix and $I_{M}\left(n\right)$ is the coefficient selection matrix.

The coefficient selection matrix $I_{M}\left(n\right)$ is a diagonal matrix with elements on the diagonal equal to 1, if a corresponding coefficient is selected for update, or to 0, if not:

(6) $$I_{M}\left(n\right)=\left[\begin{array}{cccc} i_{0}\left(n\right) & 0 & \cdots & 0\\ 0 & i_{1}\left(n\right) & \cdots & 0\\ \vdots & \vdots & \ddots & \vdots \\ 0 & \cdots & 0 & i_{L-1}\left(n\right) \end{array}\right],$$

where

(7) $$i_{k}\left(n\right)\in \left\{0,1\right\},\;\sum_{k=0}^{L-1}i_{k}\left(n\right)\leq M.$$

In each iteration, the elements on the diagonal of the selection matrix are selected as equal to 0 or 1, according to the following formula:

(8) $$i_{k}\left(n\right)=\left\{\begin{array}{c} 1\;\mathrm{if}\;k\in I_{M(n)}\\ 0\;\mathrm{otherwise} \end{array}\right.;$$

where $I_{M(n)}$ denotes a set of the filter weight indexes that define the coefficients to be updated in the *n*-th iteration; the number of the elements in such set is equal to, or less than *M*. At least one of the sets has the number of elements equal to *M*, which is the maximum number of filter weights updated in any iteration.

Similarly, the leakage matrix $G_{M}\left(n\right)$ is a diagonal matrix and is defined as:

(9) $$G_{M}\left(n\right)=\left[\begin{array}{cccc} g_{0}\left(n\right) & 0 & \cdots & 0\\ 0 & g_{1}\left(n\right) & \cdots & 0\\ \vdots & \vdots & \ddots & \vdots \\ 0 & \cdots & 0 & g_{L-1}\left(n\right) \end{array}\right],$$

where

(10) $$g_{k}\left(n\right)\in \left\{1,\gamma \right\}.$$

Specifically, the matrix contains elements equal to 1 in the rows corresponding to 0-s in the $I_{M}\left(n\right)$ matrix, and a selected value of the leakage γ in the rows corresponding to 1-s in the coefficient selection matrix. In this way, the leaky version of the PU-LMS algorithm in Equation (5) applies leakage in a particular iteration only to those coefficients, which are updated in this iteration. Otherwise, the advantage of processing of only a subset of parameters would be ruined.

The difference between particular PU LMS algorithms is in the way the elements of the coefficient selection matrix (and the leakage matrix) are selected, as described below.

### 3.1. Sequential LLMS

The sequential LLMS organizes the filter weights into several subsets, which are then updated *sequentially* [10]. The number of subsets the filter is partitioned with can be calculated as:

(11) B=⌈L/M⌉;

where ⌈·⌉ denotes the ceil operation. The way the filter weights are assigned to a specific subset depends on the implementation. For example, consider a simple case with L=2M, e.g., the algorithm updates half of the filter weights in each sampling period. In such a case, it is possible to update the parameters with even indexes in one iteration, and the parameters with odd indexes in the next iteration. The weights selection matrix (6) can than be written as:

(12) $$\begin{array}{cc} I_{M}\left(n\right)= & \mathrm{diag}\left(1,0,1,0,1,0\ldots \right)\\ I_{M}\left(n+1\right)= & \mathrm{diag}\left(0,1,0,1,0,1\ldots \right). \end{array}$$

Another possibility is to update the first half of the weights in one iteration, and the second half in the next iteration:

(13) $$\begin{array}{cc} I_{M}\left(n\right)= & \mathrm{diag}\left(1,1,1\ldots 0,0,0\ldots \right)\\ I_{M}\left(n+1\right)= & \mathrm{diag}\left(0,0,0\ldots 1,1,1\ldots \right). \end{array}$$

Other choices are also possible.

The Sequential LLMS algorithm operates with a constant step size ($\mu \left(n\right)=\bar{\mu }$). A property of this LLMS algorithm is that the subsets created by particular partitioning of the weights vector and defined by the weights selection matrix are processed in a sequence. Therefore, after *B* iterations, all the weights are updated.

### 3.2. Sequential LNLMS

The sequential LLMS algorithm can also be used with the step size normalization. Similarly to the (full update) NLMS algorithm, the normalization can either be considered as a modification of the LLMS algorithm, with the step size dependent on the energy of the input signal, or may be developed formally as a solution of a constrained optimization problem [14]. Regardless of the approach, the resulting algorithm operates with a variable step size given by:

(14) $$\mu \left(n\right)=\frac{\bar{\mu }}{u^{T}\left(n\right)u\left(n\right)}.$$

Otherwise, the algorithm is identical to the sequential LLMS. We will refer to this algorithm as the sequential Leaky Normalized LMS (sequential LNLMS).

### 3.3. Stochastic LLMS Algorithm

It may be proved that the sequential LLMS algorithm is permanently unstable (regardless of the step size) for some input signals, e.g., for cyclostationary signals [6]. Therefore, the stochastic LMS algorithm was developed, which selects the subsets of the weights to be updated on a random basis. The random selection should be organized in a way that selects each of the subsets with equal probability. For such algorithm, the set of filter weight indexes from Equation (8) is defined as:

(15) $$P\left(M(n)=k\right)=\pi_{k},\;k=1,\ldots B,\;\sum_{k=1}^{B}\pi_{k}=1;$$

where P denotes the probability density function of an independent random process M(n), and B=⌈L/M⌉.

Please note that it is necessary to randomize the subset selection rather than the decision if to update every individual parameter. This assures that the complexity reduction is attained during each sampling period. Please note also that the computational cost of the Stochastic LMS algorithm is slightly higher than in case of the Sequential LMS, as the random selection mechanism requires more time than the sequential selection. For further details, refer to [15].

Provided the simulation time is long and the random selection is uniform, the stochastic LLMS algorithm properties and behavior are similar to the sequential LLMS, with the exception of avoiding instability for some signals. Therefore, the algorithm will not be included in the experiments presented below.

### 3.4. M-Max LLMS Algorithm

One of the simplest ideas about the data-dependent PU algorithms could be to select *M* those entries of the input vector u(n), which result in the largest magnitude changes of the filter weights. The algorithm thus constructed is called the M-max LMS algorithm [16], and its weights selection matrix (6) entries can be defined as:

(16) $$i_{k}\left(n\right)=\left\{\begin{array}{cc} 1\; & \mathrm{if}\;|u\left(n-k+1\right)|\in \max_{1\leq l\leq L}\left(|u\left(n-l+1\right)|,M\right)\\ 0 & \mathrm{otherwise} \end{array}\right.;$$

where $\max_{l}\left(u_{l},M\right)$ denotes a set of *M* maxima of the elements $u_{l}$ [6]. In case of this algorithm, the step size is constant: μ(n)=μ.

The M-max LLMS algorithm requires ranking (sorting) of the input vector elements, based on their absolute values. Therefore, the time savings this algorithm offers are smaller than in case of the data-independent algorithms. On the other hand, computationally efficient sorting algorithms can be applied to lessen the sorting computational burden [11].

### 3.5. M-Max LNLMS Algorithm

Similarly to the sequential LNLMS, the idea of M-max PU can be applied together with the step size normalization. In this case, the weights selection matrix is defined identically as in the case of M-max LLMS algorithm (Equation (16)), and the step size is normalized as in Equation (14).

### 3.6. Selective LLMS Algorithm

The M-max LNLMS algorithm has a subtle drawback: it uses the power of the whole input vector to normalize the step size in spite of the fact that only some of its elements are used for the actual weight update. To correct this issue, it is possible to calculate the normalized step size as:

(17) $$\mu \left(n\right)=\frac{\bar{\mu }}{u^{T}\left(n\right)I_{M}\left(n\right)u\left(n\right)}.$$

This results in the algorithm known as the selective LLMS algorithm.

It is also possible to *derive* the selective LLMS algorithm as a result of instantaneous approximation of the Newton’s method, or as an application of the minimum disturbance principle [6]. Both the derivations result in the update Equation (5) and the step-size normalization given by Equation (17).

The difference between the selective LLMS and the M-max LNLMS algorithms may appear to be small, but it will be shown that it results in substantial differences in performances of both the algorithms.

### 3.7. One Tap Update LLMS Algorithm

As previously mentioned, the selective LLMS algorithm with the extreme choice of M=1 will be considered separately and will be referred to as the One Tap Update LLMS (OTU LLMS). By combining Equations (5) and (17) and using M=1, we notice that the algorithm is given by:

(18) $$w\left(n+1\right)=G_{1}\left(n\right)w\left(n\right)+\frac{\bar{\mu }}{u_{\max}^{2}\left(n\right)}I_{1}\left(n\right)u\left(n\right)e\left(n\right),$$

where $u_{\max}\left(n\right)=\max\left|u\left(n\right)\right|$ denotes the maximum absolute value in the input vector at a discrete time *n*. The maximum absolute value $u_{\max}\left(n\right)$ is assumed to be unique, and if it is not, a single element corresponding to one of the maximum absolute values in the input vector is selected for the update at a random basis.

Remembering that $w_{k}\left(n\right)$ denotes the *k*-th filter coefficient, the algorithm may be expressed as:

(19) $$w_{k}\left(n+1\right)=\left\{\begin{array}{cc} \gamma w_{k}\left(n\right)+\frac{\bar{\mu }e\left(n\right)}{u(n-k)}, & \mathrm{if}\;|u(n-k)|=\max|u(n)|\\ w_{k}\left(n\right), & \mathrm{otherwise} \end{array}\right..$$

To conclude, this simple update algorithm developed by Douglas [17], updates only one coefficient—the coefficient that corresponds to the input sample with the maximum absolute value.

Considering the shift structure of the input vector, the *maxline* algorithm [11] can be used for finding its maximum absolute value. The worst case computational complexity of this algorithm is L+1 comparisons, and only one multiplication, one division and one addition per iteration. However, it has been shown in [11] that the average number of comparisons, in case of a random input data with the uniform distribution, is approximately equal to 3 and **does not increase with the filter length**. Of course, calculation of the filter output still requires *L* multiplications and L−1 additions.

### 3.8. Computational Power Requirements and Implementation

The computational demands of the discussed algorithms are given in Table 1. To calculate the requirements for the data-dependent algorithms, it was assumed that the input vector is a tap-delayed vector, and the *sortline* algorithm can be used to sort this vector. Therefore, the cost of the sorting was assumed to be $2\lceil \log_{2}L\rceil +2$ comparisons. For further details, refer to [6], but also note that the leakage requires additional multiplications in the number equal to the number of updated weights (*L* for full update and *M* for PUs).

Practical implementation of the leaky PU algorithms is similar to the implementation of other LMS-family algorithms. The architecture of modern microprocessors, especially those which are referred to as “digital signal processors”, is optimized to perform the MAC operation [18]; therefore, the implementation of the actual filtering (expressed by the summation on the right-hand side of Equation (4)), is usually highly effective. The practical implementation of the adaptation, on the other hand, is quite different from straightforward implementation of Equation (5) in a programming language. The equation suggests the use of two matrices ($I_{M}$ and $G_{M}$), which are in fact omitted, because they are only used to define indexes of those filter weights, which are selected for updates by a particular PU algorithm. Thus, the algorithm loops through all the selected indexes and performs updates by application of the leakage γ and subtraction of the product of the step size μ(n), error e(n) and a corresponding value from the input vector u(n). For more details on the whole digital signal processing path consult, e.g., [19].

As was already mentioned, the data-dependent algorithms require sorting or calculation of a maximum value of a vector. Practical aspects of implementation of such algorithms are discussed in [6].

## 4. Multichannel Active Noise Control Problem

Noise is one of major civilizational issues and therefore is of great scientific attention [20]. Passive noise control techniques have been used for years and are very effective when it comes to attenuation of high-frequency noise. Unfortunately, low-frequency noise is more difficult to attenuate passively; for this reason, active noise control (ANC) methods were developed [18,21].

Structural ANC systems are the systems that apply active control to the noise-emitting structure rather than to an additional loudspeaker [22,23]. These methods have earned a lot of attention among the scientific community due to the fact that they allow to achieve a global noise reduction, hard to obtain using different methods [24,25]. Unfortunately, structural ANC belongs to the group of the most demanding algorithms, when it comes to the computational power [19]. This is due to the fact that controlling even a simple structure usually requires more than one sensor and actuator, even if the structure is only a single panel or wall. To present a more realistic example, consider a system controlling the noise propagating from a device with a cuboid enclosure. The numbers of walls to control differs from 3 (for the device positioned in a corner of a room) to 5 (for the device standing far from the walls of a room). Suppose the device is positioned close to a wall (but not in a corner), so that 4 devices walls are to be controlled, and each is controlled using 4 actuators. Suppose there are 5 error sensors in the room—the number of active filters to consider will be equal to 80 (4 walls times 4 actuators times 5 error sensors). The hardware demands of such a multichannel system operating with a reasonable sampling frequency and even short filter lengths (e.g., 128) are enormous; therefore, only adaptation algorithms with low computational power demands can be considered for such a case and the alike. Partial updates belong to the group of such algorithms, and we will demonstrate by the means of simulations that leaky PU LMS algorithms can be successfully used.

Several techniques can be used to lower the computational demand. The most intuitive way is to use shorter adaptive filters, but this attitude may result in poor attenuation results for noises more complicated than sinusoidal signals. Another attitude is to use a computational power saving algorithm. There are a few such algorithms, e.g., the switched error algorithm [26]; however, the partial updates can also be used, as reported in previous research [4,7] and this communication. Two experimental setups, based on existing, real laboratory stands, will be used to demonstrate by simulations that PU algorithms with leakage are particularly well-suited for structural ANC.

### 4.1. Experimental Setup 1: Active Casing

**Figure 1:**
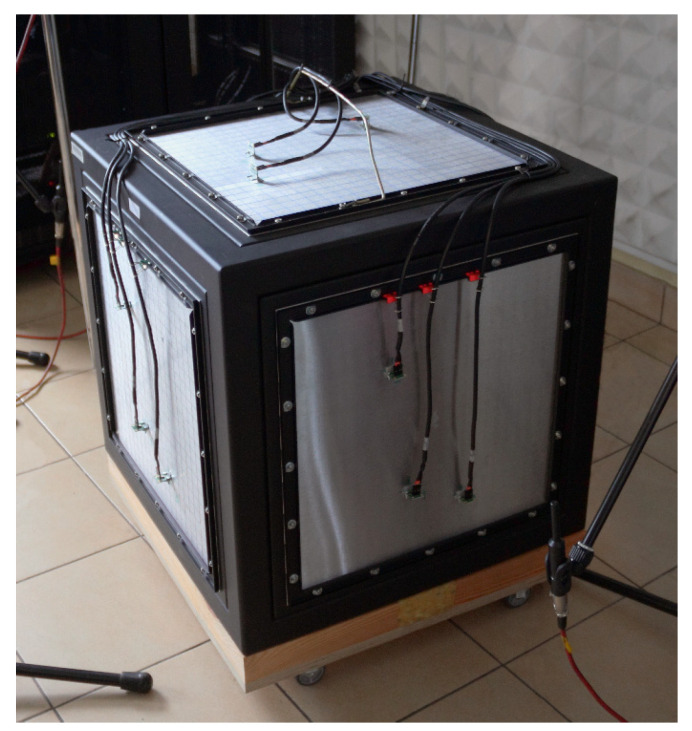
The rigid casing used in Experimental Setup 1.

The first experimental setup consists of an active casing with rigid corners and 1 mm thick aluminum plates, with 420 × 420 mm dimensions, as presented in Figure 1. Each plate was equipped with three electrodynamic Monacor EX-1 5 Watt actuators, mounted in carefully selected positions to improve controllability of the system. The casing was placed in the laboratory room a little distance off the walls so five sides of the casing were available and an error sensor (microphone) was positioned in front of each side [22].

The above setup consists of 15 actuators and 5 error microphones; thus, the number of secondary paths to consider is 75. The models of those secondary paths were identified in the form of finite impulse response (FIR) filters with 256 parameters. The identification experiments were performed using white noise as an excitation and using the sampling frequency of 2 kHz. In addition, a loudspeaker was placed inside the casing and the primary paths were identified as well, in the same form. Finally, a reference signal path was identified between the loudspeaker and a microphone placed inside the casing.

**Figure 2:**
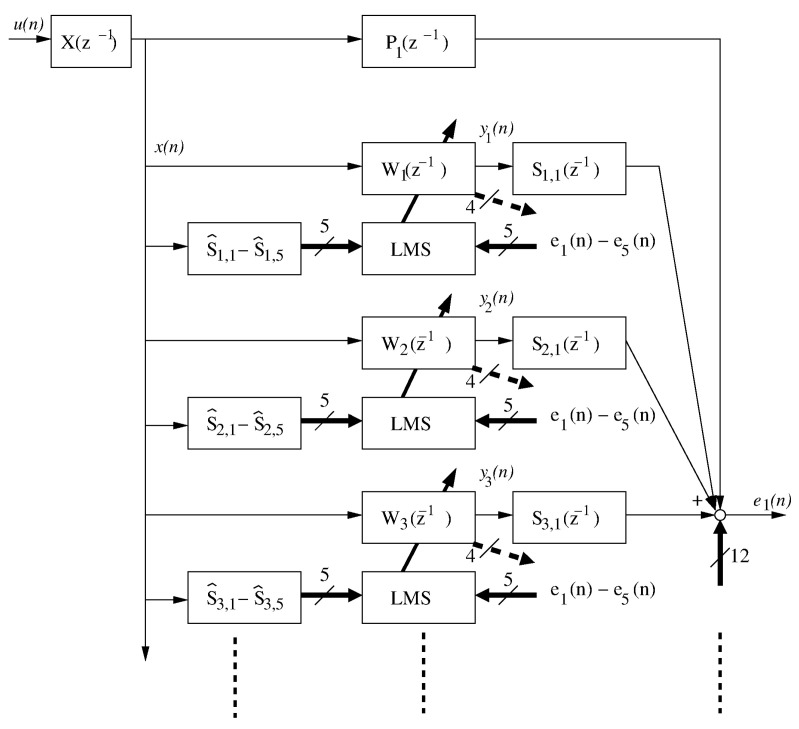
Block diagram of the simulated active casing system.

The identified transfer functions (TF) were used to design a simulation system allowing for rapid testing of adaptation algorithms. A part of the block diagram of this system, associated with one of the casing walls, is presented in Figure 2. According to this figure, the noise to be attenuated u(n) is filtered through the reference path TF $X(z^{-1})$, to produce the reference signal, x(n). The reference signal forms the input to the primary path TF $P_{j}\left(z^{-1}\right)$, where *j* is the corresponding error microphone number. This reference signal forms the input signal to the control filters—three such filters, $W_{1}\left(z^{-1}\right),W_{2}\left(z^{-1}\right)$, and $W_{3}\left(z^{-1}\right)$, associated with the front plate are presented in Figure 2. Each control filter produces the output $y_{j}\left(n\right)$, which is then filtered with five different secondary path TFs; one of these TFs for each of the presented filters is visible in the figure. The secondary path TFs are denoted as $S_{i,j}\left(z^{-1}\right)$, where *i* is the actuator number (i∈{1…15}), and *j* is again the error microphone number. Three secondary path TFs, i.e., $S_{1,1}\left(z^{-1}\right)$, $S_{2,1}\left(z^{-1}\right)$, and $S_{3,1}\left(z^{-1}\right)$, are presented in Figure 2, while the remaining twelve belong to the other error signal paths. In consequence, each error signal is a sum of 16 signals: one primary signal and 15 signals coming from the 15 actuators.

All 5 error signals are used by each of the adaptation algorithms, denoted as LMS in Figure 2 [18,27]. In addition, each of the adaptation algorithms uses 5 reference signals, each filtered through a different secondary path TF estimate, e.g., ${\hat{S}}_{1,1}$–${\hat{S}}_{1,5}$ in case of the algorithm associated with the $W_{1}\left(z^{-1}\right)$ control filter. The simulation system is then fairly advanced and requires 165 filtrations and 15 adaptations in each simulation iteration.

**Figure 3:**
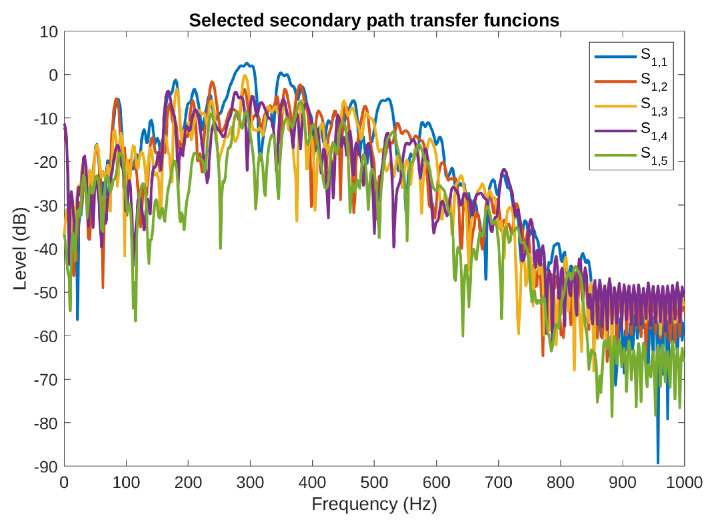
Secondary path transfer functions from the first actuator on the front plate to all the five error microphones.

Exemplary frequency characteristics of the secondary path TFs from one of the actuators mounted on the front plate to all the five error microphones are illustrated in Figure 3. From the figure, it can be noticed that the magnitude of the presented TFs is similar in the average; however, large variations of magnitude for all the five TFs are observed at several particular frequencies. For example, $S_{1,4}\left(z^{-1}\right)$ and $S_{1,5}\left(z^{-1}\right)$ have a very low magnitude at 115 Hz, the difference between $S_{1,5}\left(z^{-1}\right)$ and $S_{1,3}\left(z^{-1}\right)$ is around 40 dB at this frequency.

**Figure 4:**
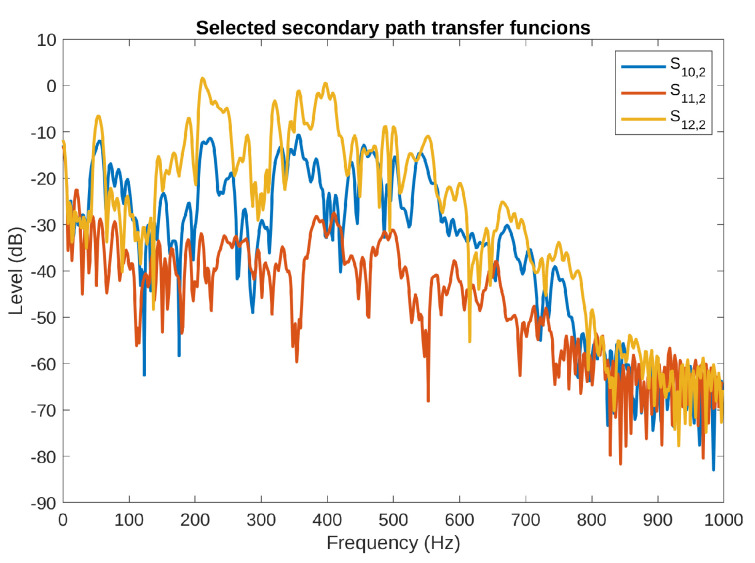
Secondary path transfer functions from the top plate actuators to the left plate error microphone.

Figure 4 presents the frequency characteristics of the secondary path TFs from the three top plate actuators to one of the error microphones. In this case, the magnitudes of the presented TFs differ much more from each other than those presented in Figure 3. Moreover, similar differences were observed for the TFs to the other four error microphones. Special attention should be paid to the $S_{11,y}\left(z^{-1}\right)$ TF, which has the lowest average magnitude among all the secondary path TFs.

Both the above mentioned figures suggest that the identified models are accurate and contain a variety of phenomena present in real environment, contrary to some other, simplified models (e.g., TF of 16 order) used in simulations presented in the literature. Moreover, the secondary path TF estimates (${\hat{S}}_{x,y}\left(z^{-1}\right)$ in Figure 2) used in the simulations presented below were not perfect. Instead, all the models were truncated down to 128 parameters, and each impulse response parameter was disturbed with a small, random value.

The simulations described in this section, unless otherwise specified, were performed using the following setup. The sampling frequency was assumed to be 2 kHz. The noise signal was generated as a sum of two sinusoids, with frequencies 112 and 191 Hz, shifted in phase by π/4. A white noise sequence was also generated of the same length and was filtered with a band-pass filter with pass band between 100 and 500 Hz, resulting with a sequence for which the variance was equal to 0.4. Such noise was added to the two-sine signals to present a more realistic signal. The active filter length was set at 256. Three different step sizes were used: 1 ×$10^{-5}$ for the algorithms with a constant step size, 8 ×$10^{-3}$ for the algorithms with the step size normalization, except for the selective LLMS (including the One Tap Update LLMS), for which the step size was equal to 4 ×$10^{-3}$. The leakage factor used was 0.999999, except for the eleventh active filter—the filter associated with the second sensor on the top plate—for which the leakage factor was 0.99999. The step sizes and leakage factors were determined by trial and error, with an intention to achieve fast adaptation and robust performance (the robust performance was confirmed by simulation of combinations of different frequencies, within the band 100–300 Hz). The simulations were repeated 50 times, with different white noise sequences, to achieve smoother mean square error (MSE) curves.

### 4.2. Simulation Results

**Figure 5:**
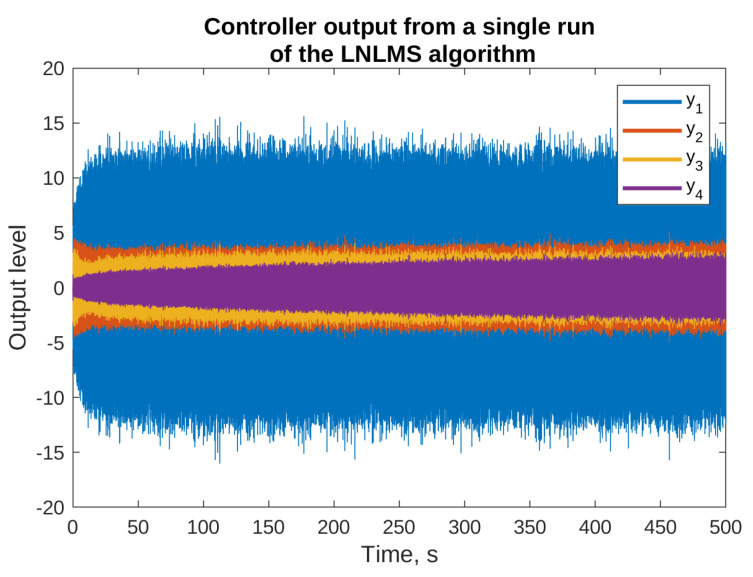
Selected outputs of the adaptive filters for the simulations of the NLMS algorithm without the leakage.

**Figure 6:**
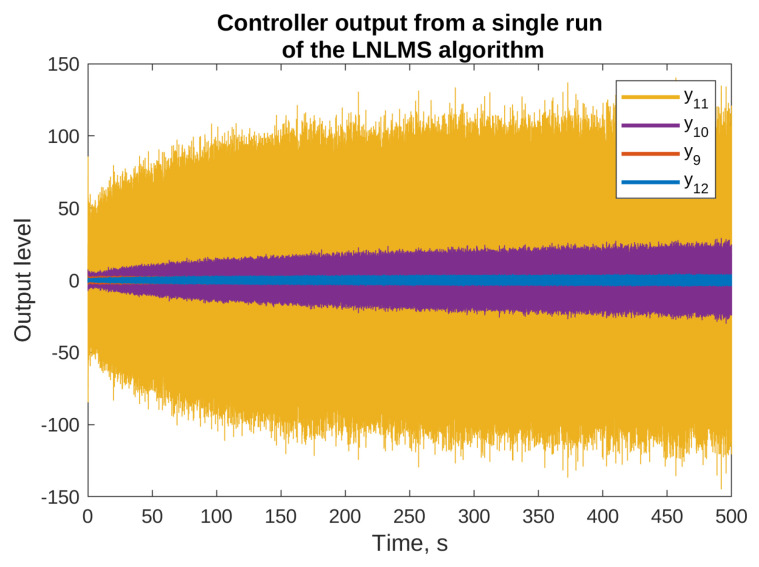
Selected outputs of the adaptive filters for the simulations of the NLMS algorithm without the leakage.

The reasons for using the algorithms with leakage are presented in Figure 5 and Figure 6, where the results of simulation of the NLMS algorithm without leakage are presented. The figures present the output signals from the adaptive filters only (the final attenuation towards the end of this simulation was around 37 dB). From the figures, it is clear that some of the output signals have substantially higher levels than the others. For example, $y_{1}$ output level reaches ±15, $y_{10}$ output stays within ±20, and $y_{11}$ exceeds ±100. On the other hands, the majority of signals are bounded by ±5. It can be also noticed that some of the signals did not achieve a steady state even after 500 s of the simulation, and are still growing slowly. Both the facts suggest that the described algorithm without leakage is not suitable for practical applications, because it would probably result in exceeding the digital-to-analog converters limits, and it would probably be unstable after longer periods of operation. Therefore, all the remaining simulations presented in this paper use algorithms with leakage.

The results also suggest that a lower value of the leakage should be applied for those filters that give higher output values. Unfortunately, particular values of the leakage factor are hard to determine in a way other than by trial end error. In practical ANC applications, this is usually done based on observation of the filter parameters, which should be approximately constant in the steady state.

**Figure 7:**
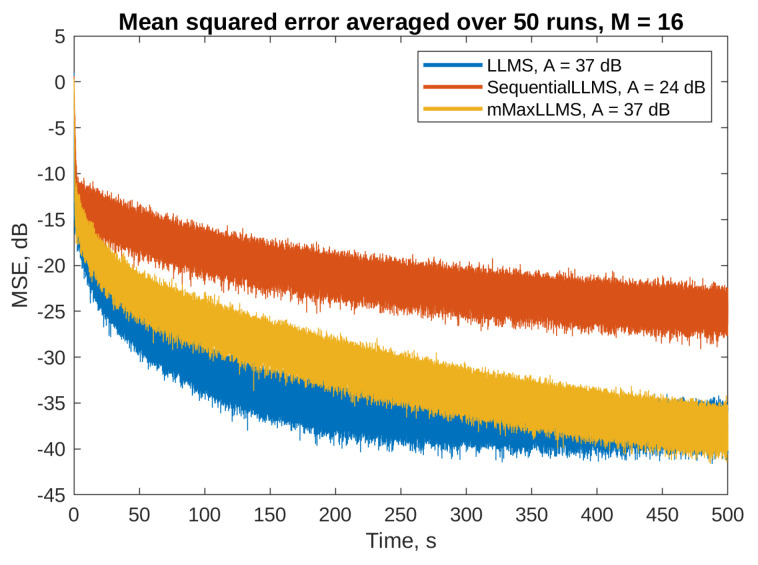
Active noise control results on the third error microphone for the algorithms without the step size normalization and *M* = 16. Final attenuation level for each algorithm is given in the legend.

**Figure 8:**
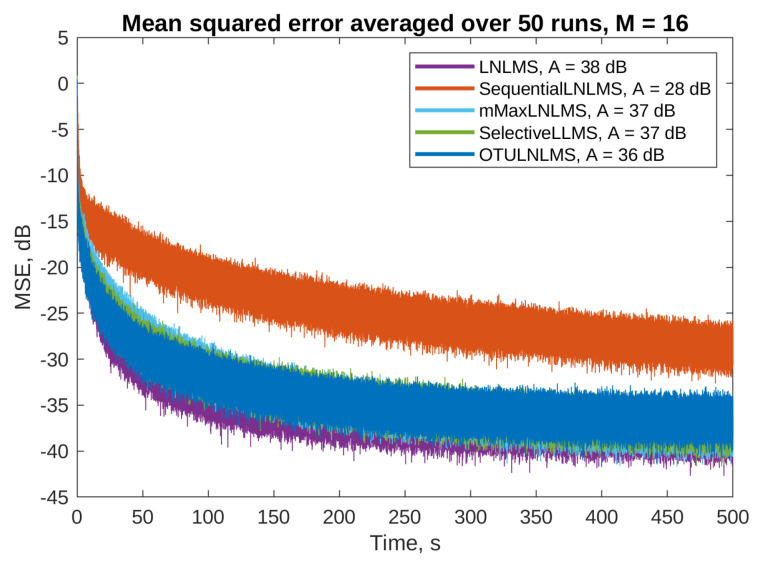
Active noise control results on the first error microphone for the algorithms with the step size normalization and *M* = 16. Final attenuation level for each algorithm is given in the legend.

Figure 7 and Figure 8 present the mean squared error (MSE) obtained on the selected error microphones during simulations of algorithms without and with the step size normalization, respectively. The PU algorithms were simulated with M=16, i.e., 16 adaptive filter parameters out of 256 were updated in each simulation step. The full update LLMS and LNLMS algorithms were added to the simulations for a reference. The attenuation level was calculated based on the MSE during the last 50 s of each simulation.

The figures show that all the algorithms are stable and converge at different speeds, reaching noticeable attenuation levels towards the end of the simulation. Among the algorithms without the step size normalization (Figure 7), the full update algorithm is the fastest to converge, and attains the highest level of attenuation; however, the M-max LLMS algorithm converges only a little slower, and attains the same level of attenuation. The sequential LLMS algorithm converges very slowly (approximately 16 times slower); therefore, it achieves only 24 dB of attenuation within the simulation window.

The algorithms with the step-size normalization (Figure 8) are capable of achieving even better performance: converge faster and achieve higher levels of attenuation. It is interesting to notice that all the PU algorithms except the sequential LNLMS algorithm show similar performance than the full update LNLMS. The selective LNLMS algorithm shows the fastest initial convergence speed and attains an attenuation level only slightly worse than the full update. However, the OTU LNLMS algorithm is the third, reaching an impressive attenuation level of 36 dB despite the fact that it updates only one filter weight in each simulation step.

The final attenuation levels obtained for all five error microphones for all the simulated algorithms are presented in Table 2. From the table, it is clear that the attenuation level calculated based on $e_{3}$ was the best, while the attenuation for $e_{5}$ was the worst. The latter fact can be explained based on the geometry of the laboratory, where the fifth microphone, corresponding to the back panel, was close to the room wall.

**Figure 9:**
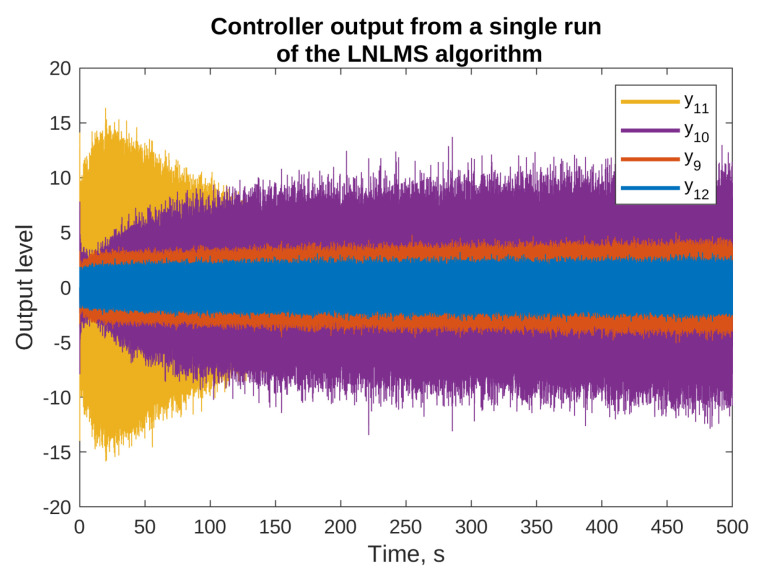
Selected outputs of the adaptive filters for the simulations of the LNLMS algorithm with leakage.

Figure 9 presents selected output signals obtained during simulation of the LNLMS algorithm, corresponding to those in Figure 6. It can be observed that introduction of the leakage prevented the output signals from reaching very high levels—this fact is especially visible in case of $y_{11}$.

**Figure 10:**
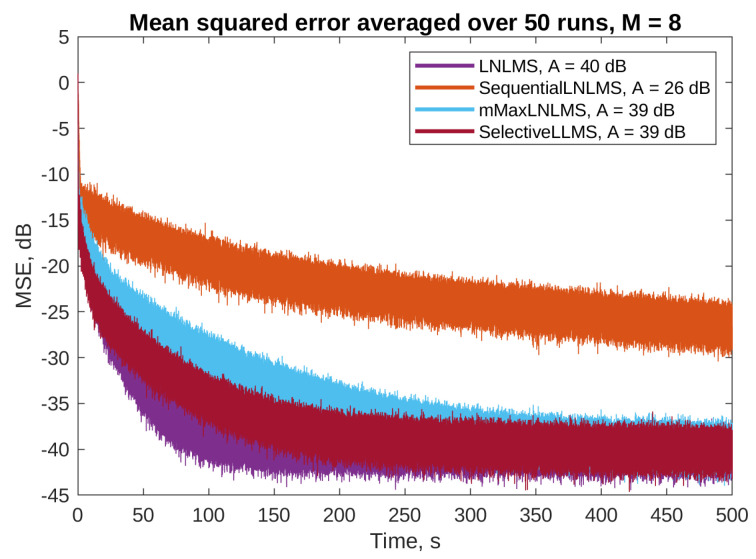
Active noise control results on the third error microphone for the algorithms with the step size normalization and *M* = 8. Final attenuation level for each algorithm is given in the legend.

Figure 10 and Table 3 present the results of simulations with M=8 (eight filter taps updated in each simulation step). The figure shows only the algorithms with the step size normalization, which show better performance. The table does not include the algorithms which do not use the *M* parameter (i.e., full update algorithms and the OTU LNLMS). It can be noticed that updating only eight parameters results in slightly worse performance, compared to simulations with M=16, but the results are still acceptable in the means of both the convergence speed and the attenuation level.

### 4.3. Experimental Setup 2: Washing Machine

**Figure 11:**
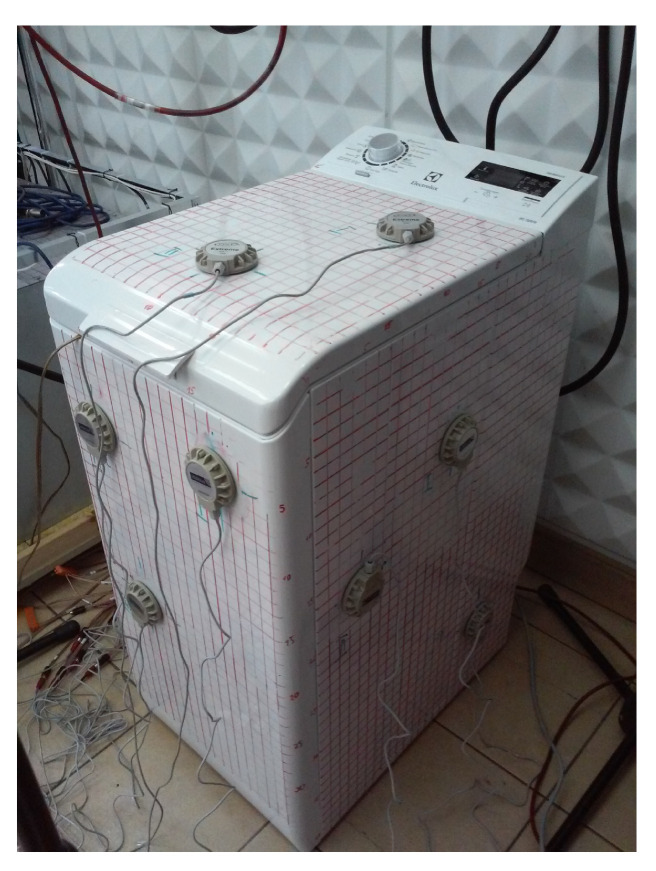
The washing machine used in the experiments.

The second experimental setup uses a commercial washing machine, as presented in Figure 11. The device was placed in the laboratory close to one of the walls; therefore, only four walls of the machine were available. Thirteen 5 W electrodynamic actuators were placed on the machine: three on each of the three side walls and four on the top wall (contrary to the Figure, which was taken before the final mount). The reference sensor was placed inside the washing machine, below the drum. The laboratory room was equipped with eight microphones, positioned in a quarter-sphere arrangement, used as error sensors (the final application will probably use other type of sensors, e.g., accelerometers attached to the device, and using the Virtual Microphone technique [28]). As was described in [22], examination of the results of attenuation on the eight error microphones allows us to determine the global attenuation effect.

Similar to the case of the first experimental setup, impulse responses of primary and secondary paths were identified, each with 256 parameter. There were eight primary paths from the reference microphone to the error sensors, and 104 secondary paths, from the 13 actuators to each of the error sensors. The same sampling frequency of 2 kHz was used. Again, the simulation system was implemented in the Matlab environment with the block diagram similar to this presented in Figure 2. This system was used to produce the results presented below.

**Figure 12:**
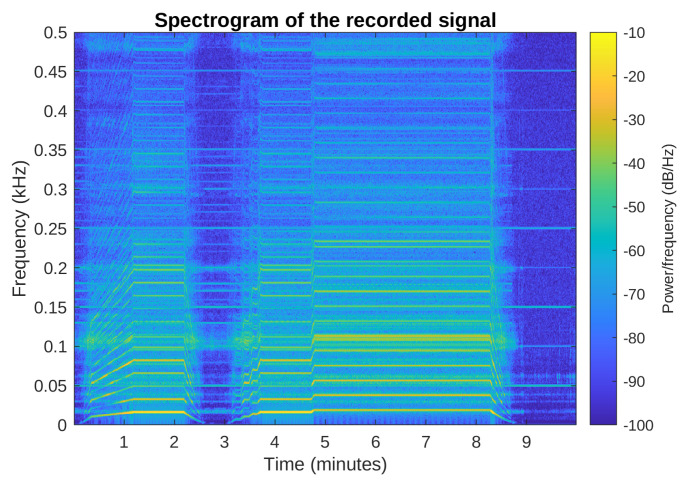
Spectrogram of the signal recorded during the washing machine final spinning phase.

The simulations were performed using a signal recorded during the washing machine spinning cycle, which is the loudest part of each washing. The spectrogram of this signal is presented in Figure 12, where the variety of the spinning signal harmonic components can be observed. For example, the spinning frequency is constant and equal to 1140 rpm between 5th and 8th minute of the cycle; therefore, the 19 Hz component and its products are easily distinguishable. Nevertheless, it should be noted that the signal is nonstationary: the washing machine increases the rotation speed slowly or even sometimes abruptly (as right before the 5th min), maintains a constant speed for an amount of time, and then slowly reduces the speed. The simulations presented below used only a part of this recording, approximately between minute 3 and 8.

The active filter length used in the following simulations was 256. Based on the results obtained with the rigid casing, it was assumed that it will be sufficient to update eight filter parameters in each iteration (M=8). Three different step sizes were used: 1 for the algorithms with constant step size, 4 ×$10^{-3}$ for the algorithms with the step size normalization, except for the selective LLMS (including the One Tap Update), for which the step size was equal to 1 ×$10^{-3}$. The leakage factor used was 0.999999 for all the filters. Again, the step sizes and leakage factors were determined by trial and error, with an intention to achieve fast adaptation and robust performance. The simulations were repeated five times without resetting the filter weights to avoid zero initial conditions, and the results of only the last run are presented below.

### 4.4. Simulation Results

**Figure 13:**
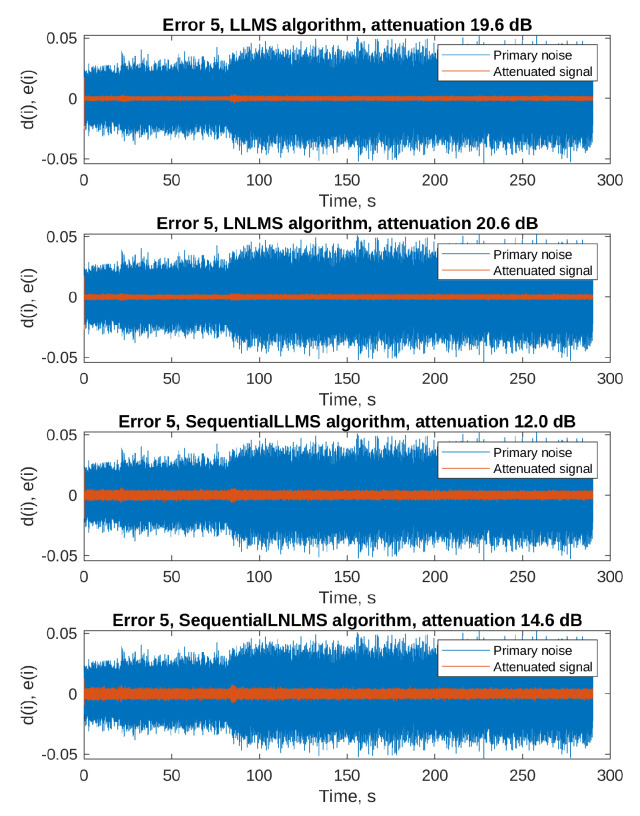
Primary noise and the attenuated signal on the error microphone 5—part 1.

**Figure 14:**
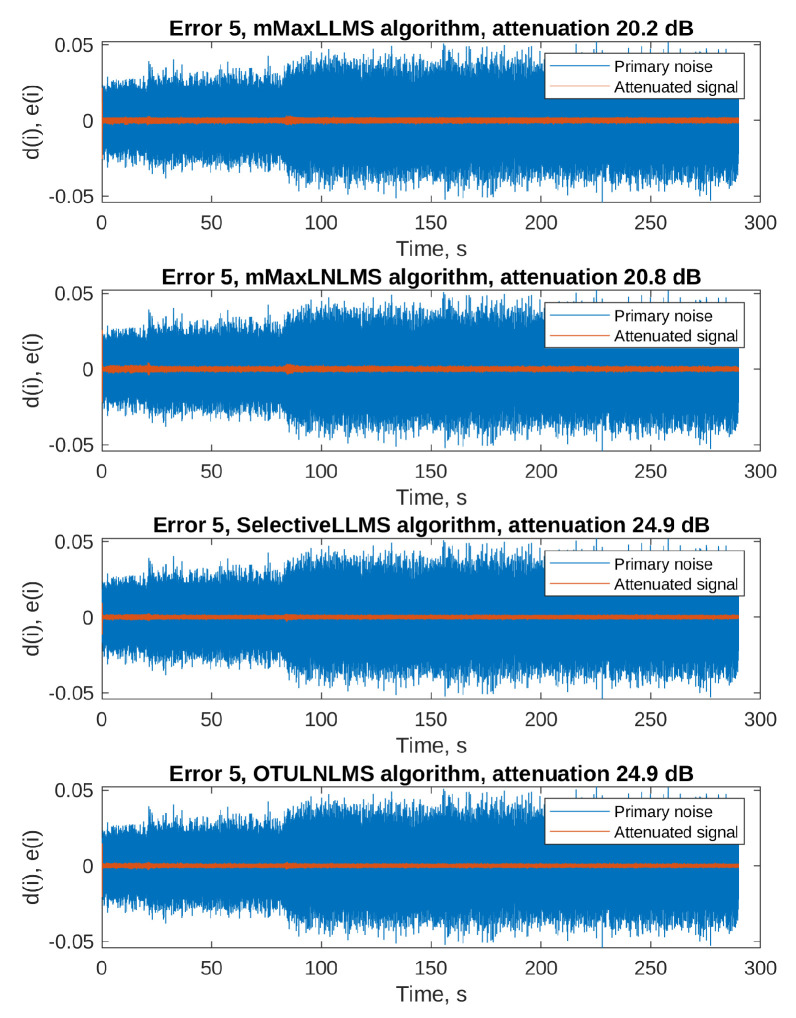
Primary noise and the attenuated signal on the error microphone 5—part 2.

The adaptation process of a filter that does not start with zero initial conditions does not produce results similar to those presented in Figure 7 and Figure 8: the MSE level is low from the beginning of the simulation, and increases only a little during the attenuated signal rapid changes. Additionally, the MSE cannot be smoothed by averaging several runs, since each single run uses exactly the same spinning cycle recording. Therefore, Figure 13 and Figure 14 present only time plots of signals simulated on a selected error microphone: the primary noise (blue) and the attenuated signal (orange). The fifth error microphone was selected because it produced the most spectacular results; however, substantial attenuation was recorded on each of the 8 error microphones. Table 4 presents the results of attenuation for every simulated algorithm on each of the error microphones. The attenuation was calculated from the ratio of the primary noise variance and the attenuated signal variance during the last 10% (29 s) of the simulation experiment.

By analyzing the results, we conclude that all the PU Leaky LMS algorithms are capable of achieving good attenuation levels, from 8 dB in the worst-case scenario, up to almost 30 dB. Of course, the sequential algorithms result in the poorest attenuation levels, but it must be remembered that these algorithms offers the best computational power savings. What can be surprising is the fact that two PU algorithms, namely the selective LNLMS and the OTU LNLMS algorithms achieved better results than the full update LNLMS. However, it must be remembered that the step size used with these algorithms was slightly different (0.004 for the full update vs. 0.001 for the PU algorithms), which might have given a little advantage to the PU algorithms. Nevertheless, the results obtained with the algorithm that updates only 1 filter weight out of 256 (OTU) are impressive.

**Figure 15:**
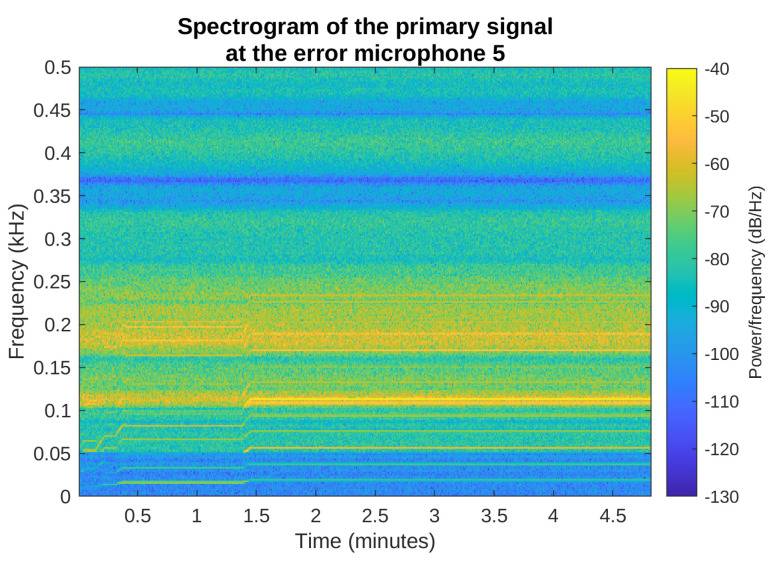
Spectrogram of the primary noise on the error microphone 5, Selective LNLMS algorithm.

**Figure 16:**
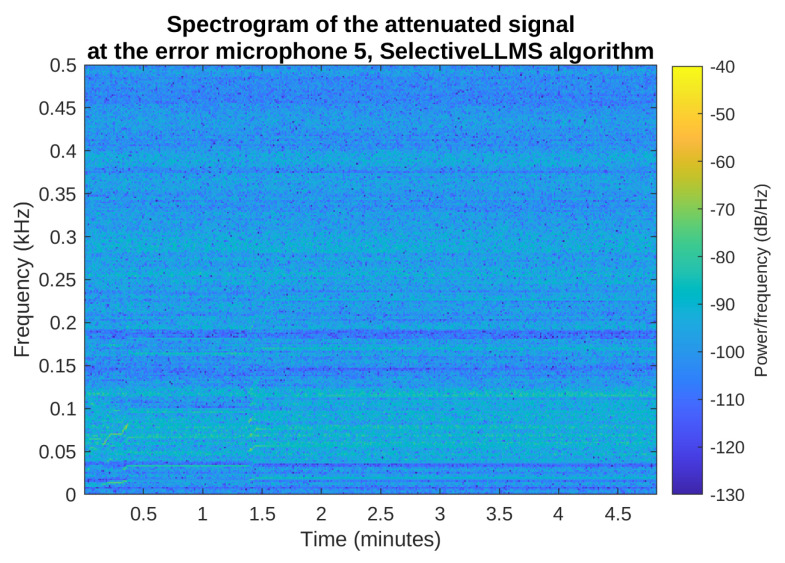
Spectrogram of the attenuated signal on the error microphone 5, Selective LNLMS algorithm.

Figure 15 and Figure 16 show the spectrograms of the primary noise and the attenuated signal in the most interesting frequency range from 0 to 500 Hz, for the selective LNLMS algorithm and the fifth error microphone. The spectrogram of the attenuated signal confirms that the attenuation is obtained during the full simulated period of time, for all the spectral components of the noise signal.

**Figure 17:**
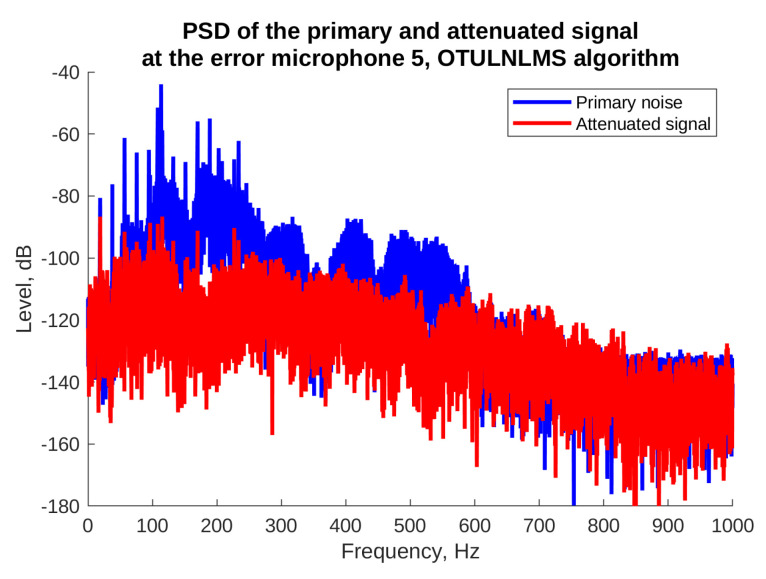
Powers spectral density without and with ANC for the microphone with the highest attenuation, OTU LLMS algorithm.

**Figure 18:**
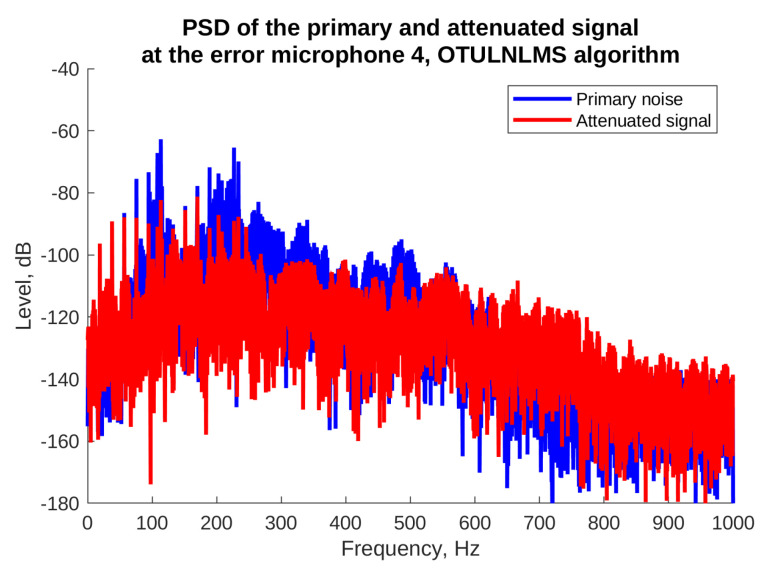
Powers spectral density without and with ANC for the microphone with the poorest attenuation, OTU LLMS algorithm.

Finally, Figure 17 and Figure 18 present power spectral densities of the noise signal and the attenuated signal during the last 10% of the simulation time, for the OTU LNLMS algorithm and error microphones where the best and the worst attenuation was obtained, i.e., $e_{5}$ and $e_{4}$. In Figure 17 we observe very good attenuation of almost all harmonic components, except for the lowest frequency component (17 Hz). Fortunately, such low frequency is already outside the normal human hearing frequency range. The figure also shows a good attenuation of broadband noise present in the recorded signal. On the other hand, Figure 18 shows that some lower frequency harmonic components were not attenuated sufficiently in the error microphone 4 location. Observe, however, that the whole level of the signals from the fourth microphone is lower than in case of the fifth microphone. Considering this, the final levels of both the attenuated signals are similar.

## 5. Conclusions

The goal of the research presented in this paper was to study usefulness of several partial updated LMS algorithms in a very demanding application to structural ANC. From a practical implementation point of view, overestimated multichannel ANC systems usually require adaptation algorithms with leakage, because leakage allows us to equalize individual actuator control efforts, thus resulting in stable adaptation and higher attenuation levels. Therefore, to allow for application of PU LMS algorithms in such systems, leaky versions of the algorithms were introduced. In case of PU algorithms, the leakage must be implemented in a way that does not ruin the computational power savings. The main contribution of this paper is the presentation of the proper attitude towards solution of this problem, and the presentation of the resulting leaky PU LMS algorithms (the sequential LLMS, sequential LNLMS, M-max LLMS, M-max LNLMS, selective LLMS and One Tap Update LLMS). The computational power savings offered by these algorithms with the respect to the full update LLMS algorithm are also presented and discussed.

To test the proposed algorithms in a rapid development environment, two simulation systems were constructed, based on the structural ANC real laboratory setups. The first system was based on an active casing with rigid walls; five panels were controlled by three actuators on each wall. The second was based on a commercial washing machine placed close to the laboratory room wall, so four washing machine walls were controlled with a total of 13 actuators. Both the systems were multichannel, with 75 and 104 secondary paths, respectively. Contrary to many other publications, the secondary path estimates used in these simulations were not perfect. The simulated noises included two sinusoids embedded in noise and a real recording of a washing machine spinning cycle.

The simulations confirmed that the proposed leaky PU algorithms can be used in structural ANC systems. All the leaky PU LMS algorithms tested were capable of providing between 8 and 40 dB of attenuation. It must be emphasized that in case of the simulated structural ANC system, the attenuation effect is global and is not concerned only with the error microphone location. Therefore, the obtained results are satisfactory and prove the leaky PU LMS algorithms have a high potential for real application.

The main disadvantage of PU LMS algorithms is possible degradation of the convergence speed, which is an inherent feature of all data-independent PUs. Therefore, data-independent algorithms should be used with caution when fast convergence is important. Data-dependent algorithms, on the other hand, do not result in the convergence speed decrease, but require the implementation of sorting of the input vector. This in turn lowers the computational power savings these algorithms offer. An interesting compromise may be the leaky One Tap Update algorithm, which instead of sorting utilizes maximum values searching—the algorithms that can be implemented in a very optimal way.

## Figures and Tables

**Table 1:** Computational complexity of the discussed PU algorithms.

Algorithm	Multiply	Add	Div	Compare
LLMS	3L+1	2L		
LNLMS	3L+2	2L+2	1	
Sequential LLMS	L+2M+1	L+M		
Sequential LNLMS	L+2M+2	L+M+2	1	
mMax LLMS	L+2M+1	L+M		$2\lceil \log_{2}L\rceil +2$
mMax LNLMS	L+2M+2	L+M+2	1	$2\lceil \log_{2}L\rceil +2$
Selective LLMS	L+2M+2	L+M+2	1	$2\lceil \log_{2}L\rceil +2$
OTU LNLMS	L+2	L+1	1	3 *

* The average number of comparisons for uniformly distributed data. In the worst-case scenario, the number of comparisons is the same as with the other data-dependent algorithms.

**Table 2:** Active casing attenuation levels obtained for each error microphone for M=16.

Algorithm	$e_{1}$	$e_{2}$	$e_{3}$	$e_{4}$	$e_{5}$
LLMS	33.6 dB	29.1 dB	37.2 dB	26.4 dB	24.8 dB
LNLMS	37.8 dB	30.0 dB	40.5 dB	28.1 dB	25.3 dB
Sequential LLMS	21.8 dB	19.8 dB	24.5 dB	18.5 dB	18.7 dB
Sequential LNLMS	28.4 dB	22.2 dB	31.4 dB	20.9 dB	20.5 dB
mMax LLMS	33.6 dB	30.2 dB	37.1 dB	26.0 dB	22.4 dB
mMax LNLMS	37.2 dB	31.3 dB	40.7 dB	28.3 dB	24.0 dB
Selective LLMS	36.9 dB	33.4 dB	39.1 dB	31.5 dB	27.1 dB
OTU LNLMS	36.2 dB	34.0 dB	38.6 dB	32.7 dB	27.7 dB

**Table 3:** Active casing attenuation levels obtained for each error microphone for M=8.

Algorithm	$e_{1}$	$e_{2}$	$e_{3}$	$e_{4}$	$e_{5}$
Sequential LLMS	19.9 dB	18.0 dB	22.2 dB	16.9 dB	16.7 dB
Sequential LNLMS	24.2 dB	18.8 dB	26.1 dB	18.3 dB	19.0 dB
mMax LLMS	31.9 dB	28.1 dB	34.7 dB	24.1 dB	20.6 dB
mMax LNLMS	35.1 dB	28.4 dB	39.4 dB	26.1 dB	21.8 dB
Selective LLMS	37.7 dB	34.4 dB	41.1 dB	32.0 dB	29.8 dB

**Table 4:** Washing machine attenuation levels obtained for each error microphone for M=8.

Algorithm	$e_{1}$	$e_{2}$	$e_{3}$	$e_{4}$	$e_{5}$	$e_{6}$	$e_{7}$	$e_{8}$
LLMS	14.0 dB	13.6 dB	17.7 dB	12.7 dB	26.5 dB	16.9 dB	21.0 dB	19.6 dB
LNLMS	14.2 dB	13.3 dB	15.4 dB	12.8 dB	26.2 dB	16.3 dB	20.9 dB	20.6 dB
Sequential LLMS	10.4 dB	9.5 dB	14.3 dB	8.3 dB	21.0 dB	12.9 dB	16.4 dB	12.0 dB
Sequential LNLMS	9.8 dB	9.8 dB	11.0 dB	8.7 dB	19.8 dB	12.1 dB	15.1 dB	14.6 dB
M-max LLMS	12.9 dB	13.2 dB	17.2 dB	12.4 dB	26.6 dB	15.9 dB	20.8 dB	20.2 dB
M-max LNLMS	13.5 dB	12.8 dB	15.2 dB	12.5 dB	26.5 dB	15.9 dB	20.7 dB	20.8 dB
Selective LLMS	15.9 dB	16.1 dB	17.2 dB	15.6 dB	29.5 dB	17.4 dB	23.1 dB	24.9 dB
OTU LNLMS	15.8 dB	16.8 dB	17.1 dB	16.0 dB	28.9 dB	17.0 dB	22.8 dB	24.9 dB

## Data Availability

The data presented in this study are available on request from the author.
